# Penile metastasis secondary to urothelial bladder cancer

**DOI:** 10.1016/j.eucr.2023.102428

**Published:** 2023-05-20

**Authors:** Maciej Dolny, Mateusz Czajkowski, Katarzyna Czajkowska, Małgorzata Sokołowska-Wojdyło, Marcin Matuszewski

**Affiliations:** a Department of Urology, Medical University of Gdańsk, Gdańsk, Poland; b Student Research Group at the Department of Urology, Medical University of Gdańsk, Gdańsk, Poland; c Department of Dermatology, Venerology and Allergology, Medical University of Gdańsk, Gdańsk, Poland

**Keywords:** Penile cancer, Metastases, Bladder cancer

## Abstract

Penile metastasis is extremely rare. The most common neoplasms that spread to the external male genital area are bladder and prostate cancer. The diagnosis usually begins with the appearance of penile symptoms. Further examination usually reveals metastasis to other organs, which worsens the patient prognosis.

We present a case report of an 80-year-old patient who was accidently diagnosed with metastatic high-grade urothelial cancer during a male circumcision. Further diagnostic process revealed a disseminated neoplastic disease.

Whole-body computed tomography (CT) scan often reveals disseminated neoplastic disease, which is the cause of high mortality in secondary penile neoplasms.

## 1 Introduction

Penile metastasis is extremely rare, despite rich vascularization and an extensive lymphatic network in the male external genital area. To date, there have only been 460 case reports of such ailments. The most frequent primary sites of penile metastasis are the bladder, prostate, rectum, kidney, testis, and lungs.^1^ The mechanism of metastasis remains unknown, but there are suggestions for direct extension or lymphatic, arterial, and venous retrograde spread. The most common clinical manifestations of penile metastasis are penile swelling, nodules, ulceration, hematuria, malignant priapism, and penile or perineal pain.^2^ Generally, in bladder cancer, there is an interval of up to several years between primary diagnosis and secondary to penile metastasis.^3^ Penile metastasis is usually a sign of disseminated disease and poor prognosis. Most patients require a multidisciplinary approach, despite survival at approximately 12 months after diagnosis.^4^ We present a case of incidentally diagnosed penile metastasis secondary to bladder cancer during circumcision surgery due to phimosis.

## 2 Case presentation

The 80-year-old male patient was admitted to the tertiary referral Department of Urology with phimosis. An unretractable foreskin was observed two years prior to admission. The patient had undergone cystoprostatectomy 15 years earlier with the emergence of a neobladder with the Studer method due to high-grade urothelial cancer with the staging of pT2N0Mx. Fifteen years between cystoprostatectomy and admission to the Department of Urology were uneventful, during which time the patient remained under clinical surveillance at the above-mentioned tertiary hospital. Moreover, a DDD pacemaker was implanted in a patient with hypertension and cardiac arrhythmia.

**Fig. 1:**
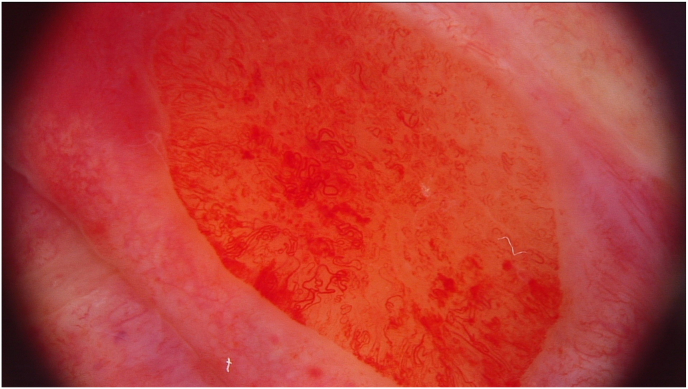
Dermatoscopic picture of polymorphic vessels on penile glans.

The patient qualified for circumcision in a tertiary hospital because of ongoing surveillance. Redness was observed after the penile glans were exposed during the surgery. Intraoperative dermoscopy revealed areas of polymorphic vessels (shown in Fig. 1). It was decided to take a biopsy of the suspicious lesions. The foreskin was then removed using the sleeve circumcision technique. The patient's postoperative period was uneventful.

**Fig. 2:**
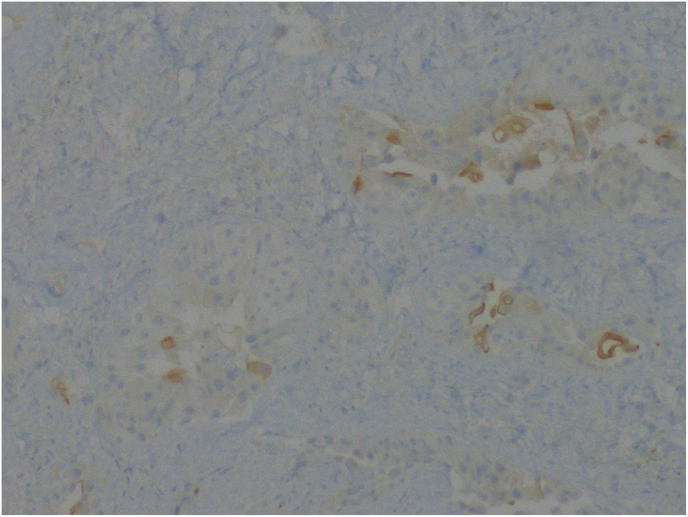
Molecular examination confirming presence of uroplakin.

**Fig. 3:**
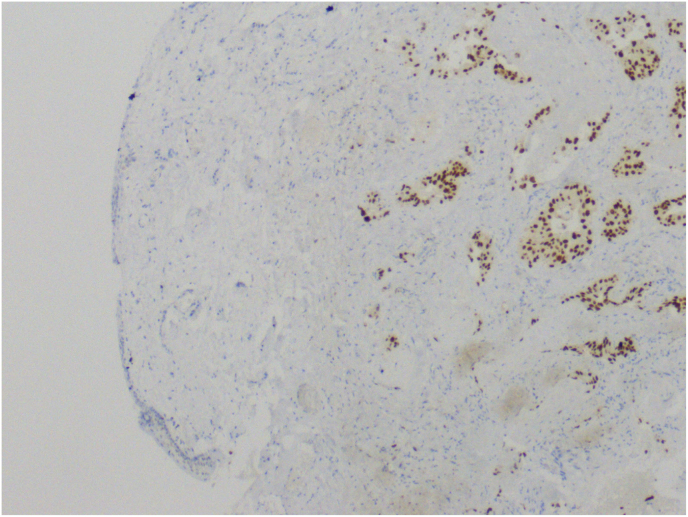
Molecular examination confirming presence of GATA 3.

Histopathological examination revealed lichen sclerosus and high-grade invasive urothelial cancer with p63 (+), uroplakin (+/-) (shown in Fig. 2), and GATA3 (+) staining (shown in Fig. 3).

Metastasis of the penile glans suggested the presence of other metastases. Thoracic and abdominal computed tomography (CT) revealed metastasis in the liver and lungs, and an ambiguous lesion in the left adrenal gland.

Palliative radiotherapy was performed due to disseminated disease. Unfortunately, a few months after circumcision, the patient died of cachexia due to disseminated disease.

## 3 Discussion

Penile metastasis was first described by Eberth in 1870.^2^ The specific pathway of metastasis is still unknown, mainly because of its rarity. The most frequent pathomechanism mentioned by the authors were retrograde venous, lymphatic, and arterial spread. Other theories include direct extension or implantation secondary to instrumentation.^2^ According to Mearini et al., who analyzed the frequency of metastasis of various cancers to the penis, bladder, and prostate, cancers were responsible for penile metastasis in 28.6% and 27.9% of cases, respectively. Other primary tumors were rectumsigmoid cancer (12.2%), kidney cancer (6.9%), lymphoma (6.8%), and lung cancer (6.2%). Retrograde venous spread is one of the most important metastatic routes because of the natural venous connection of the corpus cavernosa and penile glans with the bladder, prostate, and rectumsigmoid.^2^ Another mechanism of metastasis may be the flow of cancer cells along with urine. However, in our case, the patient underwent cystoprostatectomy, which eliminated metastasis. It is very important to verify metastasis and the primary tumor with histopathological and molecular examination to confirm the origin of the secondary tumor. In this report, the molecular examination of the lesion on the foreskin revealed uroplakin (+/-) (shown in Fig. 2) and GATA3 (+) (shown in Fig. 3), which had the same phenotype as the primary tumor in the bladder, confirming that the lesion on the foreskin was a metastasis. Usually, the first symptom is a perceptible penile mass or nodules (51%), priapism (27%), lower urinary tract symptoms (27%), pain localized to the penis and less often referred to the perineum (17%), urinary retention (13%), and skin lesions such as redness or ulceration on the penis (11%).^3^^,^^5^ Penile metastasis is regularly associated with disseminated disease. Therefore, patients usually present with symptoms in other organs affected by metastases. There are no guidelines for treatment, but the best results present surgical procedures such as local excision or total penectomy. Because of the usually associated disseminated disease, palliative chemotherapy or radiotherapy was performed. The vast majority of secondary penile tumors are symptomatic; however, in some cases, metastasis can be found at an asymptomatic stage. In this situation, fluoro-18-deoxyglucose positron emission tomography and fluorine-18-fluorochinolone positron emission tomography are used for diagnosis. In our case report, the clinical manifestations of penile metastasis were phimosis and redness of the penile. Penile metastasis, confirmed by histopathological examination, should always lead to CT, which in our case presented with metastasis in the liver and lungs. Even the surgical procedure is described as treatment, giving the best results in local metastatic tumors. In our case, only palliative radiotherapy was offered because of the absence of symptoms in the male genital area. The diagnosis of penile metastasis usually precedes the diagnosis of disseminated disease, with a poor prognosis. Most patients are offered only palliative or supportive therapy, although exceptional cases with long-term survival of 20 months or more have been reported.

## 4 Conclusion

In conclusion, penile metastases most often originate from primary bladder and prostate cancer. This phenomenon is very rare, and a whole-body computed tomography scan very often reveals disseminated neoplastic disease, which is the cause of high mortality in secondary penile neoplasms. Moreover in patient after radical cystoprostatectomy it is very important to evaluate external genital area which is often overlooked by patients.

## Funding sources

No funding

### Data availability statement

All data generated or analyzed during this study are included in this article and its supplementary material files. Further enquiries can be directed to the corresponding author.

## Author statement

Maciej Dolny: Conceptualization, Writing - Original Draft and Review & Editing Mateusz Czajkowski: Conceptualization, Resources, Writing - Review & Editing Katarzyna Czajkowska: Resources Małgorzata Sokołowska-Wojdyło: Supervision Marcin Matuszewski: Supervision.

## Declaration of competing interest

The authors have no conflicts of interest to declare.
